# Gadolinium accumulation and its biochemical effects in *Mytilus galloprovincialis* under a scenario of global warming

**DOI:** 10.1007/s11356-023-30439-2

**Published:** 2023-11-01

**Authors:** Madalena Andrade, Amadeu M. V. M. Soares, Montserrat Solé, Eduarda Pereira, Rosa Freitas

**Affiliations:** 1 Departamento de Biologia & CESAM, Universidade de Aveiro, Campus Universitário de Santiago, 3810-193 Aveiro, Portugal; 2 Departamento de Química & LAQV-REQUIMTE, Universidade de Aveiro, 3810-193 Aveiro, Portugal; 3 Departamento de Recursos Marinos Renovables, Instituto de Ciencias del Mar ICM-CSIC, Barcelona, Spain

**Keywords:** Rare-earth elements, Temperature, Oxidative stress, Metabolism, Ecotoxicity

## Abstract

Electrical and electronic equipment reaching the end of its useful life is currently being disposed of at such an alarmingly high pace that raises environmental concerns. Together with other potentially dangerous compounds, electronic waste contains the rare-earth element gadolinium (Gd), which has already been reported in aquatic systems. Additionally, the vulnerability of aquatic species to this element may also be modified when climate change related factors, like increase in temperature, are taken into consideration. Thus, the present study aimed to evaluate the toxicity of Gd under a scenario of increased temperature in *Mytilus galloprovincialis* mussels. A multi-biomarker approach and Gd bioaccumulation were assessed in mussels exposed for 28 days to 0 and 10 μg/L of Gd at two temperatures (control – 17 °C; increased – 22 °C). Results confirmed that temperature had a strong influence on the bioaccumulation of Gd. Moreover, mussels exposed to Gd alone reduced their metabolism, possibly to prevent further accumulation, and despite catalase and glutathione *S*-transferases were activated, cellular damage seen as increased lipid peroxidation was not avoided. Under enhanced temperature, cellular damage in Gd-exposed mussels was even greater, as defense mechanisms were not activated, possibly due to heat stress. In fact, with increased temperature alone, organisms experienced a general metabolic depression, particularly evidenced in defense enzymes, similar to the results obtained under Gd-exposure. Overall, this study underlines the importance of conducting environmental risk assessment taking into consideration anticipated climate change scenarios and exposures to emerging contaminants at relevant environmental concentrations.

## Introduction

The rapid technological expansion, particularly since the industrial revolution, has resulted in an enhanced social demand for electrical and electronic equipment (EEE). Consequently, an increase in generated electronic waste (e-waste) can be anticipated, due not only to a rise in EEE production, but also to a shorter lifetime of these items, reduced maintenance options, and a fast-paced replacement lifestyle (Perkins et al. 2014). Moreover, since most e-waste is incorrectly managed, disposed of, or even recycled, there is a growing concern about the release of potentially hazardous associated substances into the environment, affecting aquatic systems and their organisms (Abalansa et al. 2021; Forti et al. 2020; Widmer et al. 2005). Among these substances, rare-earth elements (REEs) are valuable and crucial components of EEE. Their importance relies on their unusual optical and magnetic characteristics, as well as their high reactivity, which allows them to be used in numerous applications (Gupta and Bose 1989; Reisman et al. 2013).

Gadolinium (Gd) in particular, is a REE employed in a variety of products, including microwaves, TV tubes, compact discs, alloys, and optical components. (Rogowska et al. 2018). Furthermore, Gd is extensively applied in the medical field for magnetic resonance imaging (MRI) as a contrast agent (Kümmerer and Helmers 2000). Contrast agents are made as chelate molecules, which are resistant to metabolic processes and can be easily excreted (Bellin 2006; Brünjes and Hofmann 2020). Due to this property, it is released unaltered in the aquatic environment and it is difficult to remove in waste-water treatment plants (WWTPs) (Künnemeyer et al. 2009; Migaszewski and Gałuszka 2016). Gd has been reported to occur in aquatic systems mainly because of anthropogenic sources derived from Gd-based contrast agents (Bau and Dulski 1996; Kulaksız and Bau 2011, 2013; Telgmann et al. 2013). That is, effluents from associated WWTPs discharge up to 200-1100 μg/L of Gd into South Africa’s rivers, while environmental concentrations ranged from 0.001-0.004 µg/L in South Africa’s and Germany’s rivers; and up to 0.181 µg/L Gd has been detected in surface waters worldwide (Rogowska et al. 2018). In Finland’s streams, Gd concentrations ranged between 0.032-207 μg/L due to the leaching of REEs from acid sulfate agriculture soils (Åström 2001). Anthropogenic Gd has also been detected in marine systems with concentrations ranging from 0.00064 up to 0.027 μg/L in San Frascisco Bay in the United States of America and from 0.14 to 0.41 μg/L at submarine outfalls from Brazil, associated with high industrialization and medical use (Hatje et al. 2016; Pedreira et al. 2018). Given the prevalence of this REE in the environment, it is essential to know its potential threat to aquatic wildlife.

Gadolinium has also been detected in several aquatic organisms, including freshwater clams *Corbicula fluminea* and mussels *Dreissena rostiformis bugensis* at concentrations between 0.004-0.110 μg/g dry weight (DW) and 0.01-0.123 μg/g DW, respectively (Merschel and Bau 2015; Pereto et al. 2020; Perrat et al. 2017) as well as in the marine scallop *Pecten maximus* at concentrations up to 0.002 μg/g DW (Le Goff et al. 2019). In recent years, this element has also been reported to cause neurotoxicity and alterations in the metabolic capacity and oxidative stress status of marine mussels, *Mytilus galloprovincialis* (Andrade et al. 2022b; Henriques et al. 2019; Trapasso et al. 2021). Other bivalves have also experienced alterations in the oxidative stress condition after Gd accumulation, including freshwater clams (*C. fluminea* and *Spisula solida*) and mussels (*D. rostriformis bugensis* and *D. polymorpha*) (Figueiredo et al. 2022; Hanana et al. 2017; Perrat et al. 2017). Moreover, Gd has disrupted the embryonic development of *Paracentrotus lividus*, *Arbacia lixula*, *Heliocidaris tuberculata* and *Centrostephanus rodgersii* sea urchins (Martino et al. 2017).

In addition to these former impacts, it is likely that abiotic factors may also have an influence on aquatic organisms and change their susceptibility to this element. One of them is climate change, undoubtedly linked to the significant increase in population and industrialization witnessed in the last century. In particular, the global mean surface temperature is expected to increase from 1.0 °C up to 5.7 °C at the end of this century depending on different CO_2_ emission scenarios (IPCC 2022). This temperature increase has already been shown to trigger a variety of physiological and cellular alterations in marine bivalves (Coppola et al. 2017; Pirone et al. 2019; Rahman et al. 2019; Velez et al. 2017). However, the responses of organisms exposed to combined abiotic factors may differ from when exposed to isolated ones. For instance, a rise in temperature may affect both the chemical speciation and bioavailability of pollutants, as well as the organisms’ susceptibility to pollutants (Noyes et al. 2009). However, in the case of emerging pollutants like REEs, this knowledge is still very scarce. As a first step in toxicity evaluation, biomarkers are often employed to assess the effects of natural and anthropogenic factors (Lomartire et al. 2021), and oxidative stress-related parameters can be regarded as early warning biochemical signals with a potential impact at higher biological levels.

Taking all the above-mentioned factors into account, the present study evaluated the biochemical changes induced in the mussel *M. galloprovincialis* when exposed to increased temperature and to an environmentally relevant concentration of Gd, but also the interaction between these two stressors. After a standard 28-days experimental assay (e.g., ASTM 2000; USEPA and USACE 1991; 1998), the biochemical responses of mussels associated with metabolism, oxidative stress, and neurotoxicity were investigated aiming to assess the influence of temperature on the toxic effects caused by Gd.

## Methodology

### Sampling and experimental conditions

Adult specimens of *M. galloprovincialis* with similar size (length: 54.4 ± 2.3 mm; width: 34.1 ± 2.6 mm) were collected at Mira’s channel in Ria de Aveiro coastal lagoon (considered an uncontaminated site at the northwest of Portugal) during low tide in November 2019. After collection, the organisms were transported to the laboratory and allowed to depurate and acclimate for 14 days. During this period, half of the organisms were under control conditions of temperature (17.0 ± 1.0 °C), salinity (30 ± 1), and pH (8.0 ± 0.1) matching those at the sampling location, whereas the other half were under the same conditions for the first week but gradually acclimated (1 °C increase per day) until reaching the targeted higher temperature (22.0 ± 1.0 °C) in the second week. The temperature was maintained constant in air-conditioned rooms. Organisms were kept in synthetic seawater (deionized water with artificial salt, Tropic Marin® SEA SALT) under constant aeration and a natural photoperiod. The synthetic seawater was renewed twice in the first week and once in the second, with the physical water conditions being re-established. Organisms were fed with AlgaMac Protein Plus (Aquafauna BioMarine®, CA, USA) at a concentration of 150,000 cells/mussel per day after the first five days of arrival and from then on, every other day.

After this acclimation period, mussels were divided into different aquaria filled with 3 L of synthetic seawater, with five individuals per aquarium and three aquaria per treatment (fifteen organisms per treatment). To evaluate Gd stability over a week period under tested treatments, positive controls—two other aquaria per treatment at the same conditions but with no mussels—were also considered. Temperature conditions during the first two weeks of acclimation were maintained over 28 days with the temperature targeted at 17.0 ± 1.0 °C to half of the organisms or 22.0 ± 1.0 °C to the other half. The high temperature of 22.0 °C was within the annual range of average temperatures (13.4–22.9 °C) in Ria de Aveiro (Coelho et al. 2014; Santos et al. 2009; Velez et al. 2015) and the global surface temperature increase projected by the IPCC in 2100 (IPCC 2022). At each experimental temperature, half of the mussels were exposed to 10 μg/L of Gd and the other half in the absence of Gd. This concentration choice was based on former bivalve studies (Hanana et al. 2017; Perrat et al. 2017) and findings from freshwater aquatic systems (Åström 2001; Olías et al. 2005; Rogowska et al. 2018). It is worth noting that literature information on Gd concentration in seawater is limited and although it is generally reported as lower, the existence of local sources of Gd contamination, resulting in higher concentrations cannot be discarded. An initial stock solution of 15 mg/L of Gd was prepared from a 10,000 mg/L commercial solution (Inorganic Ventures) with ultrapure H_2_O, and a pre-determined volume was added to each aquarium to achieve the desired concentration. A total of four treatments were tested: 1) non-contaminated organisms under control temperature (17 °C); 2) contaminated organisms under control temperature (Gd 17 °C); 3) non-contaminated organisms under increased temperature (22 °C); 4) contaminated organisms under increased temperature (Gd 22 °C).

Throughout the 28-day exposure period, mussels were fed with the AlgaMac preparation three times per week. At the end of each week, synthetic seawater was replaced and conditions (temperature, salinity, pH, and Gd concentration) re-established. To confirm the actual concentrations, samples of seawater were collected from each aquarium immediately after spiking with Gd (i.e., after adding the contaminant to water). Seawater samples were also obtained from the positive controls collected immediately after spiking, and after 24, 48, 72, and 144 h to confirm the element’s stability throughout one-week.

Organisms were sampled from the respective aquaria after the exposure period and frozen immediately with liquid nitrogen before being transported and maintained at -80 °C. No mortality was recorded during the experiment. For biochemical analyses, three frozen mussels per aquarium (nine per treatment) were considered, with the whole tissue manually homogenized with liquid nitrogen using a mortar and pestle. The homogenized tissue correspondent to each mussel was separated into five aliquots of 0.5 g fresh weight (FW) each and stored at -80 °C until further analysis. The remaining tissue of three mussels per treatment (of different aquaria) was freeze-dried and used for Gd quantification.

### Gadolinium quantification in seawater and mussel’s soft tissue

For gadolinium quantification, seawater samples were analyzed using an inductively coupled plasma mass spectrometer (Thermo ICP-MS XSeries) equipped with a Burgener nebulizer, after being diluted 20 times and acidified with HNO_3_ 2 % (for obtaining pH < 2).

For soft tissue, 200 mg of freeze-dried samples were weighted in Teflon vessels, followed by 1 mL of HNO_3_ 65 % (v/v), 2 mL of H_2_O_2_ and 1 mL of ultrapure H_2_O, being afterwards transferred to a CEM MARS 5 microwave. The microwave-aided acid digestion was performed by increasing the temperature to 170 °C for 15 min and leaving the samples at this temperature for another additional 5 min. Following the cooling of the vessels, samples were put in polyethylene flasks, which were then filled with ultrapure water to a final level of 25 mL and stored at room temperature until measurement with ICP-MS. Blanks (microwaved vessels without sample), duplicates, and the certified reference material BCR-668 (mussel tissue; 13.0 ± 0.6 μg/Kg of Gd) were used for quality control. In digested blanks, the concentration of Gd was below the methods’ limit of quantification and recovery values from the certified reference material (3 replicates) varied between 95-119 %, confirming the reliable performance of the digestion and quantification protocols.

The LOQ was 0.02 μg/L for both water and tissues (corresponding to the value of the lowest standard concentration used in the calibration curve).

### Biological responses: biochemical parameters

Biological responses were evaluated at a whole organism level through the analysis of different biochemical parameters. As indicators of metabolic capacity, the electron transport system (ETS) activity, glycogen (GLY), and total protein (PROT) levels were examined. The activities of antioxidant enzymes such as superoxide dismutase (SOD), catalase (CAT), and glutathione reductase (GR) were selected as oxidative stress defense mechanisms and the biotransformation enzymes such as glutathione *S*-transferases (GSTs) and carboxylesterases (CbEs) were considered as additional detoxification mechanisms. Lipid peroxidation (LPO) and protein carbonylation (PC) levels were used to evaluate oxidative damage and inhibition of acetylcholinesterase (AChE) activity as a sign of neurotoxicity. Nine samples per treatment (three mussels per aquaria and three aquaria per treatment) were analyzed in duplicate and considered for each parameter. Samples were extracted through homogenization with a respective buffer (ratio of 1:2, w/v) using TissueLyzer II (Qiagen) set at frequency 20 1/s for 90 sec and centrifugation at 4 °C during 20 min with the supernatant being collected at the end. Most of the parameters (GLY, PROT, SOD, CAT, GR, GSTs, CbEs, PC, and AChE) were extracted using phosphate buffer 50 mmol/L containing 1 mmol/L ethylenediaminetetraacetic acid, 1 % (v/v) Triton X-100 and 1 mmol/L dithiothreitol at pH 7.0 with samples being centrifuged at 10,000 *g*. For LPO, samples were extracted with 20 % (w/v) trichloroacetic acid and equally centrifuged at 10,000 *g*. In the case of ETS, samples were extracted with 100 mmol/L Tris-HCl with 15 % (w/v) polyvinylpyrrolidone, 0.153 mmol/L magnesium sulfate and 0.2 % (v/v) Triton X-100 at pH 8.5 and centrifuged at 3,000 *g*. Each biochemical parameter was measured using the methods outlined in Andrade et al. (2021).

### Data analyses

#### Bioconcentration factor

The bioconcentration factor (BCF) was calculated using the Arnot and Gobas (2006) equation, which was defined as the ratio of total Gd concentration in mussels' tissue to total Gd concentration measured in water after spiking.

#### Statistical and multivariate analyses

A non-parametric permutational analysis of variance with a one-factor design (Gd concentration and temperature) was used through the PERMANOVA add-on in PRIMER v6 (Anderson et al. 2008) for hypothesis testing of the biochemical data (n=3, corresponding to 3 aquaria) obtained for each parameter measured (ETS, GLY, PROT, SOD, CAT, GR, GSTs, CbEs, LPO, PC, and AChE). The data set was subjected to a square root transformation, and a resemblance matrix was created using the Euclidean distance, since it is sensitive to different units. The Euclidean distance data matrix was assessed individually using type III sums of squares and unrestricted permutation of raw data (9999 permutations). The null hypothesis tested of “mussels exposed to different treatments (17 °C, Gd 17 °C, 22 °C and Gd 22 °C) showed no significant differences among them” was considered. Significant differences (*p* < 0.05) in the figures were represented by different letters.

An ordination analysis by Principal Coordinates (PCO) was also performed using all the biochemical data. For this, the Euclidean distance similarity matrix was simplified by using the different treatments (17 °C, Gd 17 °C, 22 °C, Gd 22 °C) to calculate the distance between centroids. Vectors relating to biochemical descriptors were placed as new variables superimposed on the PCO graph using the Spearman correlation with a correlation > 75 %, considering its robustness to outliers and non-normally distributed data often included in biochemical data.

#### Integrated biomarker response

Data obtained from the biochemical parameters were combined using the integrated biomarker response index version 2 (IBRvs2), based on Beliaeff and Burgeot (2002) and modified by Sanchez et al. (2013), to illustrate the overall biochemical responses in mussels under the different treatments. This was achieved by comparing the deviation between biochemical parameters from each treatment (Gd 17 °C; 22 °C; Gd 22 °C) to those determined in the control (17 °C). To minimize variance before calculating the IBR index, a log transformation (*Y*_*i*_) was initially used, where *Y*_*i*_ = log (*X*_*i*_/*X*_*0*_), with *X*_*i*_ standing for individual biomarker data from exposure treatment and *X*_*0*_ for mean reference data from the control treatment. The general mean (*μ*) and standard deviation (*σ*) of Y_i_ were considered, and Y_i_ was then standardized using the formula *Z*_*i*_ = (*Y*_*i*_ - *μ*) / *σ*. Then, to establish a basal line centered on 0 and reflect the variations of the parameters along this line, the biomarker deviation index (*A* = *Z*_*i*_ - *Z*_*0*_) was estimated. IBRvs2 was then determined to equal IBRvs2 = |*A*|.

For the IBRvs2 calculations, all the reported biochemical parameters were considered. The biomarker deviation index findings were shown as a star plot, with the area outside of the REF line (the line that corresponds to 17 °C treatment used as reference data) representing biomarker induction and the area inside the REF line representing biomarker inhibition. In the graphical representation, the obtained IBRvs2 values are also shown with higher values indicating higher biochemical responsiveness of the treated mussels. Values were analyzed in the context of the overall responses given by the final IBRvs2 model.

## Results

### Gadolinium concentration in seawater and mussel’s tissue

The concentration of Gd in non-contaminated treatments was consistently below the limit of quantification (LOQ < 0.02 μg/L). The concentrations of Gd in the seawater soon after spiking were 9.70 ± 0.19 μg/L at control temperature and 10.10 ± 0.55 μg/L at increased temperature, both close to the targeted nominal concentration (10 μg/L) with a maximum deviation of 3 %.

Water samples aimed to test the stability over time of Gd in seawater were collected from the aquaria at 0, 24, 48, 72, 144, and 168 h after Gd spiking. Under both tested temperatures (Table 1), the coefficients of variations were below 15 % for each treatment, being the concentration of Gd confirmed stable over at least one week and demonstrating that temperature did not influence the bioavailability of this element.

**Table 1:** Gadolinium (Gd) concentration (μg/L) measured at two different temperatures in seawater from positive controls (aquaria without mussels) at 0, 24, 48, 72, 144 and 168 h after spiking

Time (h)	Gd (μg/L)	
	17 °C	22 °C
0	10.0	10.2
24	8.7	9.3
48	8.5	9.0
72	8.6	9.0
144	8.4	8.7
168	8.2	8.6

Gadolinium was detected in all mussel tissues, independently of the treatment. Non-contaminated mussels had values of 0.015 ± 0.002 μg/g dry weight (DW) at control temperature and 0.045 ± 0.012 μg/g DW at increased temperature scenario. In Gd-exposed mussels the concentrations were 0.126 ± 0.008 μg/g DW at control temperature (17 °C) and 0.150 ± 0.008 μg/g DW at 22 °C, with significant differences among treatments (Table 2). At increased temperature, the BCF (15.6 ± 0.5 L/Kg) was significantly higher than values found at the control temperature treatment (13.0 ± 0.6 L/Kg) (Table 2).

**Table 2:** Gadolinium (Gd) concentration (μg/g dry weight) in mussels soft tissues and BCF (L/Kg) after 28 days of exposure at two different temperatures and two different concentrations of Gd (0 μg/L and 10 μg/L). Different letters represent significant differences among tested concentrations, n = 3

Treatment	Mussels tissues (μg/g dry weight)	BCF (L/Kg)
17 °C	0.015±0.002^a^	-
Gd 17 °C	0.126±0.008^b^	13.0±0.6^A^
22 °C	0.045±0.012^c^	-
Gd 22 °C	0.150±0.008^d^	15.6±0.5^B^

### Biological responses

#### Metabolic capacity

**Fig. 1:**
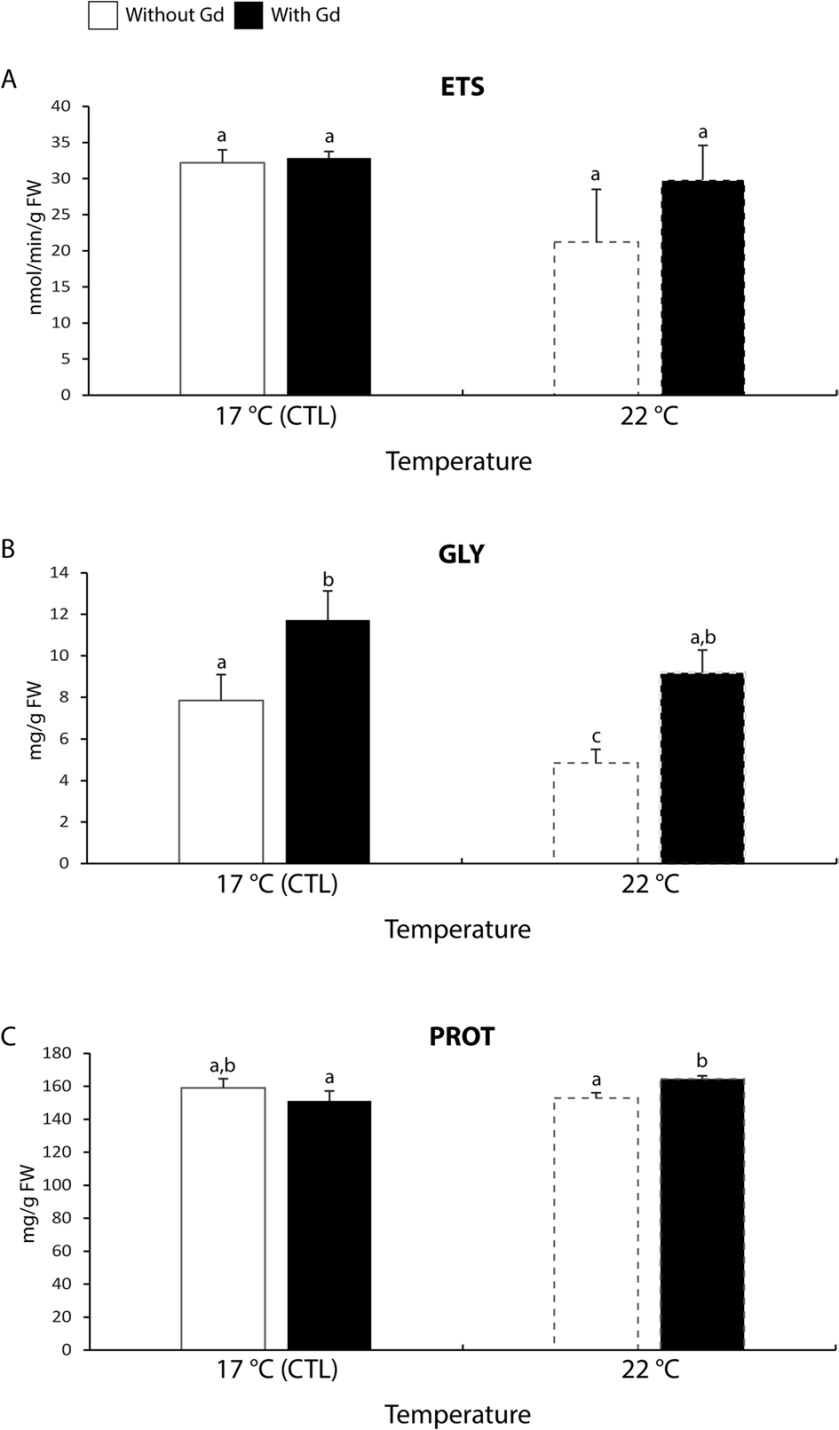
**A**: Electron transport system (ETS); **B**: Glycogen (GLY) content; C: Protein (PROT) content in *Mytilus galloprovincialis* exposed to different temperatures (17 °C and 22 °C) in the absence and presence (0 and 10 μg/L) of Gd for 28 days. Results are means with standard deviations. Significant differences (*p* < 0.05) among all four treatments are identified with different lowercase letters. FW represents the mussels’ fresh weight measured in g

The ETS activity did not differ significantly among treatments (Fig. 1A).

Regarding GLY content, mussels exposed to Gd showed about 1.5-1.9 times significantly higher content than non-contaminated ones, regardless of the test temperature (Fig. 1B). Non-contaminated mussels kept at elevated temperature showed between 1.6-2.4 times significantly lower GLY content compared with values observed in the other treatments (Fig. 1B).

The PROT content was shown to be 1.1 times significantly higher in mussels exposed to Gd under increased temperature in comparison to the ones exposed to Gd at 17 °C or non-contaminated mussels at 22 °C (Fig. 1C).

#### Antioxidant and biotransformation enzymes

**Fig. 2:**
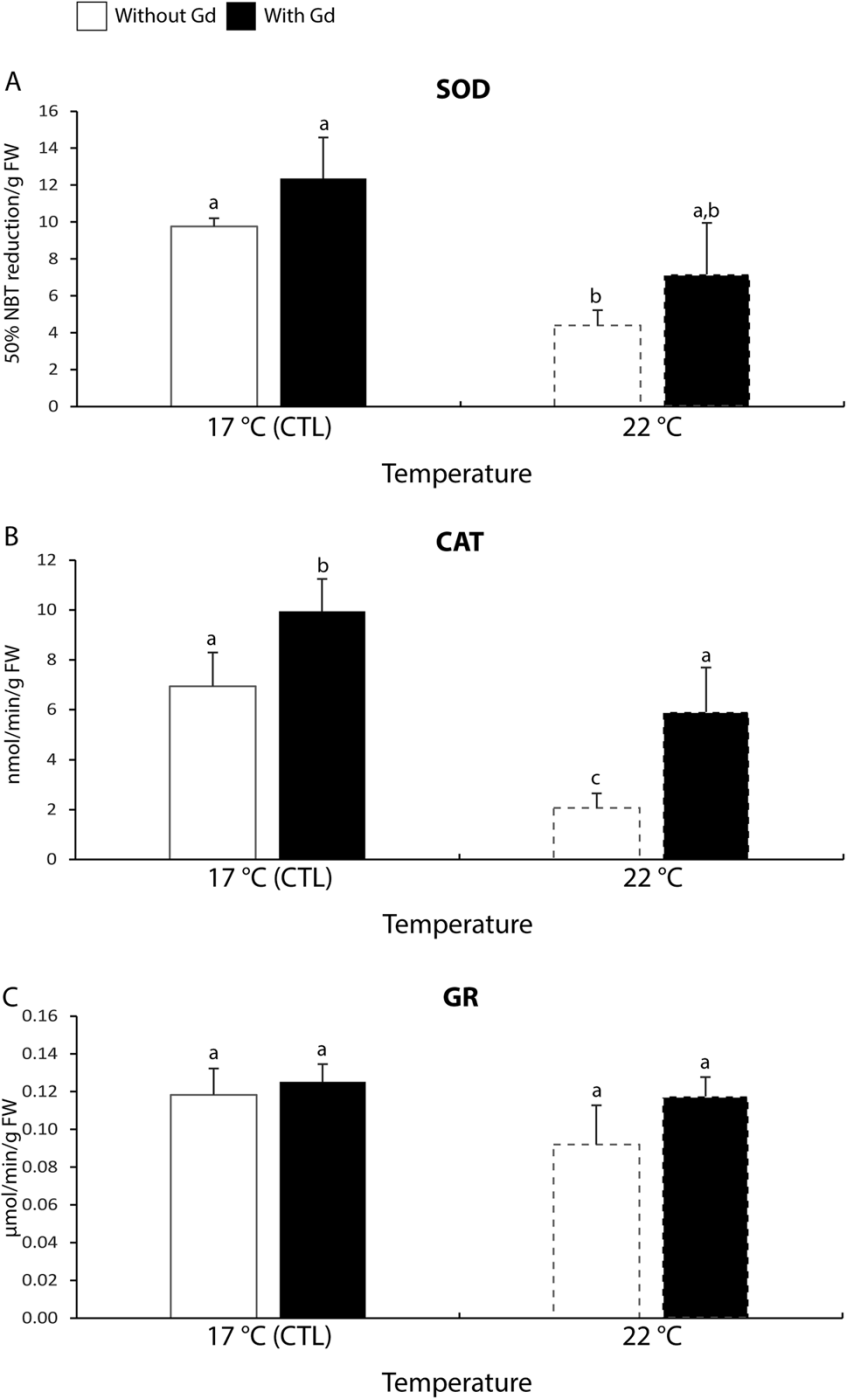
**A**: Superoxide dismutase (SOD) activity; **B**: Catalase (CAT) activity; C: Glutathione reductase (GR) activity in *Mytilus galloprovincialis* exposed to different temperatures (17 °C and 22 °C) in the absence and presence (0 and 10 μg/L) of Gd for 28 days. Results are means with standard deviations. Significant differences (*p* < 0.05) among all four treatments are identified with different lowercase letters. FW represents the mussels’ fresh weight measured in g

Concerning SOD activity, non-exposed mussels at 22 °C showed 1.7-2.2 times significantly lower activity in comparison to the ones at 17 °C (Fig. 2A).

The CAT activity displayed a similar pattern to SOD, with significant differences (1.4 times at 17 °C and 2.9 times at 22 °C) between contaminated and non-contaminated mussels at both temperatures (Fig. 2B).

The activity of GR showed no significant differences among treatments (Fig. 2C).

**Fig. 3:**
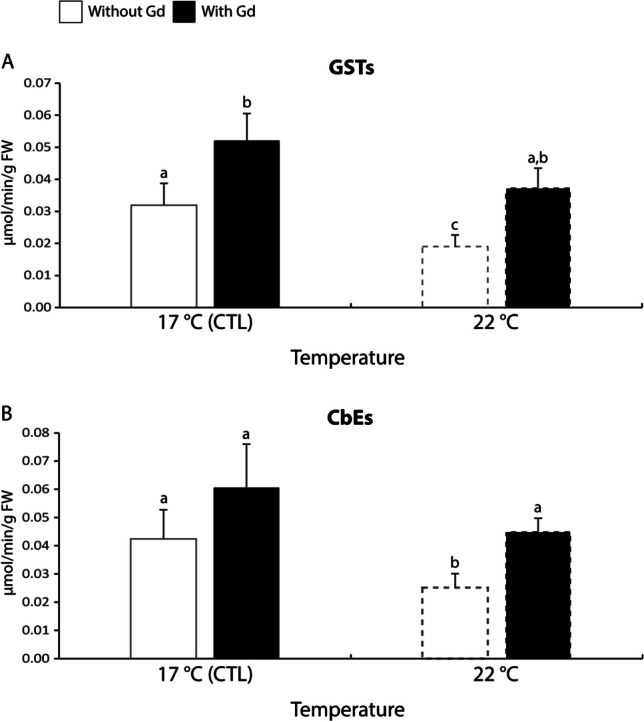
**A**: Glutathione *S*-transferases (GSTs) activity; **B**: Carboxylesterases (CbEs) activity in *Mytilus galloprovincialis* exposed to different temperatures (17 °C and 22 °C) in the absence and presence (0 and 10 μg/L) of Gd for 28 days. Results are means with standard deviations. Significant differences (*p* < 0.05) among all four treatments are identified with different lowercase letters. FW represents the mussels’ fresh weight measured in g

 Regarding GSTs activity, at control temperature, mussels exposed to Gd exhibited 1.6 times significantly higher activity than non-contaminated ones. Non-contaminated mussels subjected to 22 °C exhibited between 1.6-2.7 times significantly lower GSTs activity compared to values from all the other treatments (Fig. 3A).

In terms of CbEs activity, although the pattern of response was similar to GSTs, only non-contaminated mussels at increased temperature had their activity significantly reduced (1.6-2.4 times) compared to the other treatments (Fig. 3B).

#### Oxidative damage

**Fig. 4:**
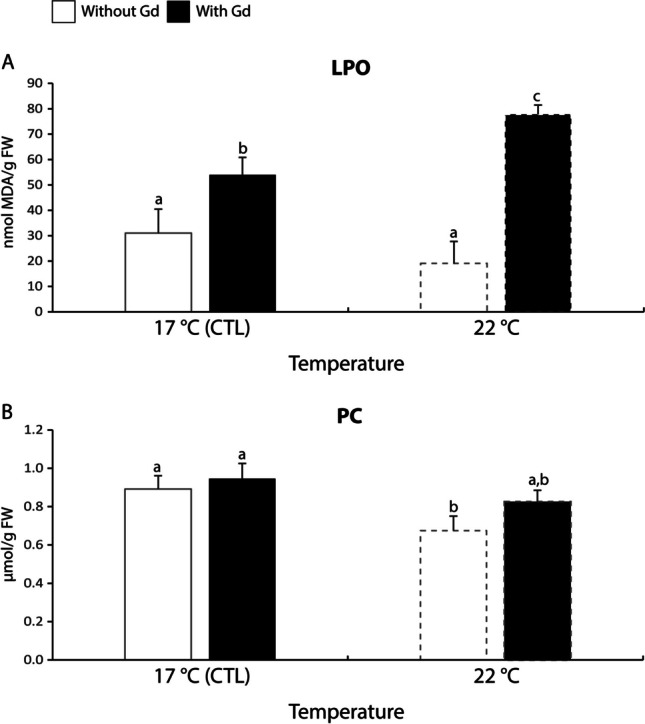
**A**: Lipid peroxidation (LPO) levels; **B**: Protein carbonylation (PC) levels in *Mytilus galloprovincialis* exposed to different temperatures (17 °C and 22 °C) in the absence and presence (0 and 10 μg/L) of Gd for 28 days. Results are means with standard deviations. Significant differences (*p* < 0.05) among all four treatments are identified with different lowercase letters. FW represents the mussels’ fresh weight measured in g

The LPO levels significantly increased between 1.7 and 4 times in mussels exposed to Gd independently of the temperature tested compared to their non-contaminated counterpart, being those Gd-exposed at 22 °C the ones with the highest LPO levels (Fig. 4A).

Concerning PC levels, non-contaminated mussels maintained at 22 °C showed significantly lower (1.3-1.4 times) values with respect to the ones under control temperature regardless of Gd presence (Fig. 4B).

#### Neurotoxicity

**Fig. 5:**
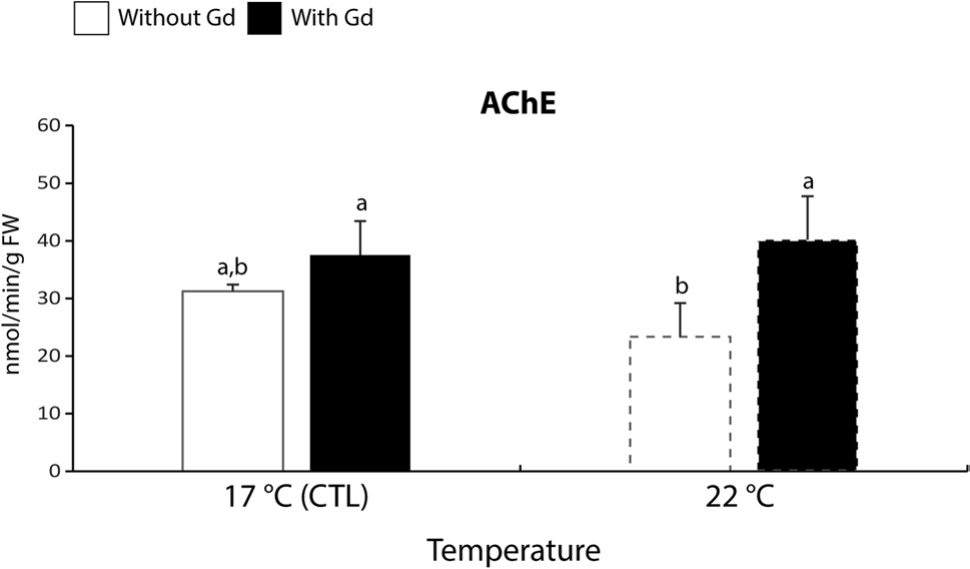
Acetylcholinesterase (AChE) activity in *Mytilus galloprovincialis* exposed to different temperatures (17 °C and 22 °C) in the absence and presence (0 and 10 μg/L) of Gd for 28 days. Results are means with standard deviations. Significant differences (*p* < 0.05) among all four treatments are identified with different lowercase letters. FW represents the mussels’ fresh weight measured in g

The activity of AChE was 1.6-1.7 times significantly lower in non-contaminated mussels exposed to 22 °C in comparison to the ones contaminated at 17 and 22 °C (Fig. 5).

### Multivariate analysis

**Fig. 6:**
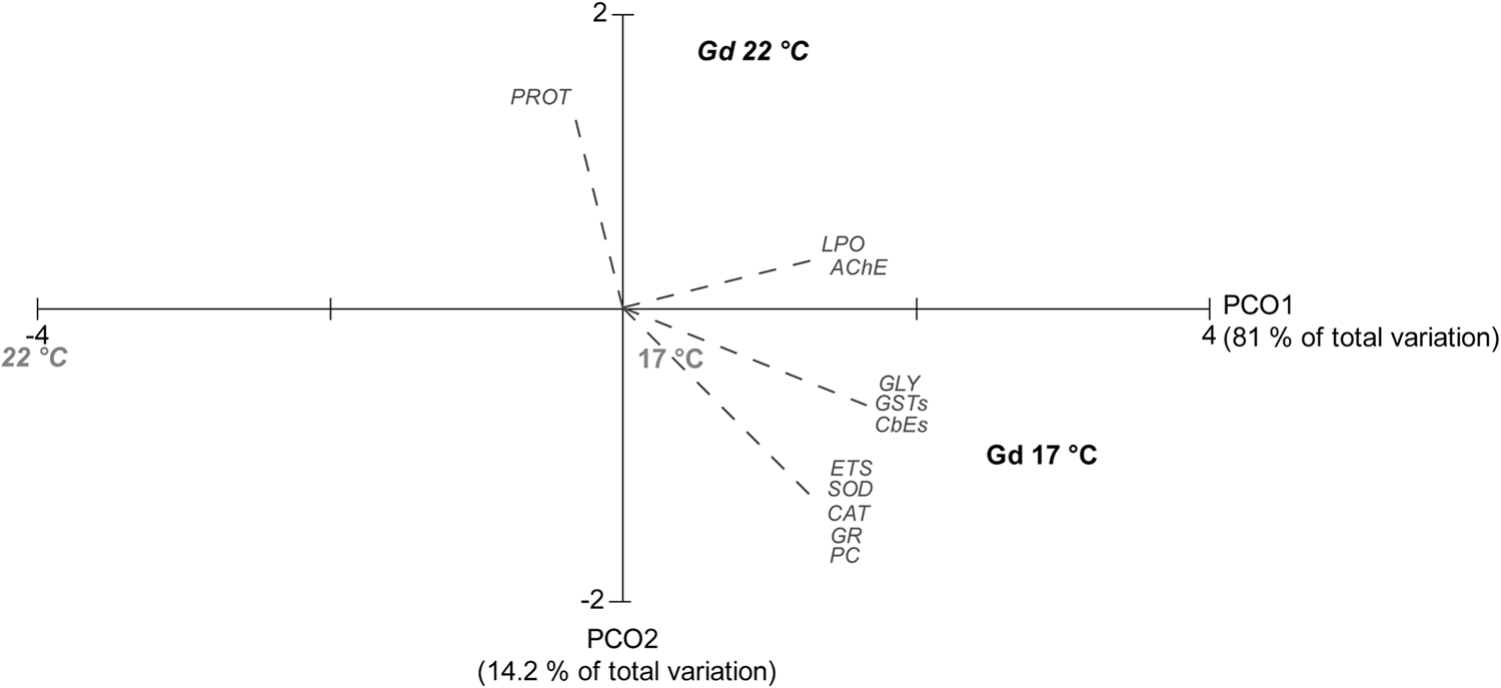
Centroids ordination diagram (PCO) based on the tested conditions and biochemical markers measured in *Mytilus galloprovincialis*. The following conditions are presented: 17 °C and 22 °C (non-contaminated organisms under different temperatures of 17 °C and 22 °C) and Gd 17 °C and Gd 22 °C (organisms exposed to 10 μg/L of Gd under different temperature of 17 °C and 22 °C). Spearman correlation vectors are superimposed as supplementary variables, namely biochemical data (*r* > 0.75): ETS = Electron Transport System, PROT = Protein, SOD = Superoxide Dismutase, CAT = Catalase, GSTs = Glutathione *S*-Transferases, CbEs = Carboxylesterases, LPO = Lipid Peroxidation, PC = Protein Carbonylation, AChE = Acetylcholinesterase

The first principal component (PCO1) of the PCO analysis (Fig. 6) represented 81 % of the total variance among treatments, it primarily separated contaminated mussels at the lowest temperature (Gd 17 °C) on the positive side of the axis from uncontaminated mussels at the increased temperature on the negative side (22 °C). The second component, PCO2 explained 14.2 % of the total variance distinguishing mussels exposed to Gd and increased temperature (Gd 22 °C) on the positive side from the ones exposed to Gd at control temperature (Gd 17 °C) on the negative side. The biochemical descriptors superimposed on the PCO1 revealed a strong positive correlation (*p* > 0.75) among all parameters with the exception of PROT and the contaminated organisms under control temperature (Gd 17 °C). This was a direct result of increased activities and levels at this treatment and the lower activities of these parameters in higher temperature (22 °C) which was on the opposite side of the axis. As for PCO2, the organisms under the combination of increased temperature and exposure to Gd, showed an especially strong positive correlation with PROT (*p* = 0.8) with higher values at this treatment, while also negatively correlating with the antioxidant enzymes, ETS and PC (*p* = -0.8).

### Integrated biomarker response

**Fig. 7:**
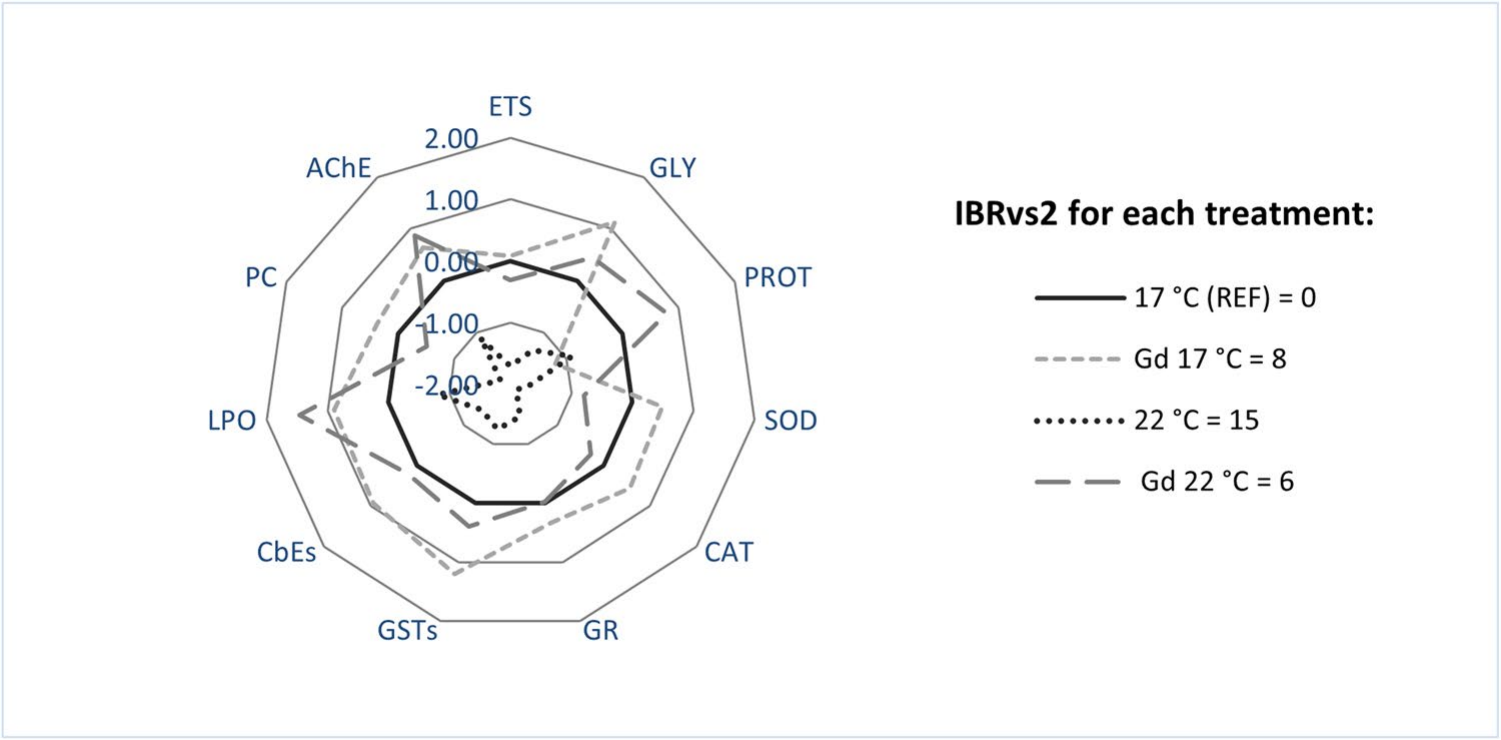
Integrated Biomarker Response (IBRvs2) index considering all biochemical parameters used on *Mytilus galloprovincialis* after exposure to different temperatures (17 °C and 22 °C) in the absence and presence (0 and 10 μg/L) of Gd for 28 days. The biomarker quantified for uncontaminated organisms under 17 °C were considered for the reference line (REF). ETS = Electron Transport System; GLY = Glycogen; PROT = Protein; SOD = Superoxide Dismutase; CAT = Catalase; GR = Glutathione Reductase; GSTs = Glutathione *S*-Transferases; CbEs = Carboxylesterases; LPO = Lipid Peroxidation; PC = Protein Carbonylation; AChE = Acetylcholinesterase

In IBRvs2, the greater values (IBRvs2 = 15) reached were those seen in uncontaminated mussels at the increased temperature (22 °C), indicating a greater impact at this temperature. The star plot clearly illustrated that this treatment resulted in lower values for all biochemical indicators, which contributed to this high score (Fig. 7). The results also showed that Gd-exposed organisms at both temperatures (Gd 17 °C and Gd 22 °C) scored similar IBRvs2 values (IBRvs2 = 8 and 6, respectively). As determining features, variations in LPO showed to be a common factor being especially relevant at the increased temperature treatment (Table 3). Nevertheless, Gd-exposed organisms at control temperature showed a slightly higher IBRv2 value associated to PROT, GLY and GSTs as these parameters were more determinant (higher inhibition or increase) at this treatment (Table 3). Between Gd treatments at the two tested temperatures, most of the enzymes analyzed showed a greater positive contribution, indicating a higher level of influence, under the control condition (17°C) (Table 3).

**Table 3:** Biomarker deviation index (A) estimated for each evaluated parameter in mussels exposed to two different temperatures and two different concentrations of Gd (0 μg/L and 10 μg/L) after 28 days. As reference, the values are normalized in relation to those obtained at 17 °C. ETS = Electron Transport System; GLY = Glycogen; PROT = Protein; SOD = Superoxide Dismutase; CAT = Catalase; GR = Glutathione Reductase; GSTs = Glutathione *S*-Transferases; CbEs = Carboxylesterases; LPO = Lipid Peroxidation; PC = Protein Carbonylation; AChE = Acetylcholinesterase

Biomarker deviation index (A)	Gd 17 °C	22 °C	Gd 22 °C
ETS	0.08	-1.7	-0.31
GLY	1.14	-1.34	0.45
PROT	-1.19	-0.91	0.79
SOD	0.49	-1.74	-0.79
CAT	0.56	-1.87	-0.27
GR	0.33	-1.46	-0.02
GSTs	1.2	-1.24	0.4
CbEs	0.94	-1.38	0.2
LPO	0.89	-0.82	1.46
PC	0.38	-1.83	-0.5
AChE	0.63	-1.14	0.86

## Discussion

### Concentration of Gd in mussels under different temperature scenarios

The present findings demonstrated the capacity of mussels to accumulate Gd in their tissues after 28 days of water-borne exposure to 10 μg/L of Gd. The accumulation of Gd has already been documented in other bivalves. For instance, the freshwater clam *Corbicula fluminea* and the mussel *D. rostriformis bugensis*, exposed to Gd concentrations of 1 and 10 μg/L for 28 days, exhibited tissue concentrations ranging from 0.002 to 0.078 μg/g DW (Perrat et al. 2017). The freshwater mussels *Dreissena polymorpha* exposed to Gd at 10, 50, 250, and 1250 μg/L also for 28 days resulted in Gd tissue concentrations of approximately 0.43, 1.35, 3.18 and 44.97 μg/g wet weight (WW), respectively (Hanana et al. 2017). Comparing the values obtained in the present study with those from other bivalves under similar Gd concentrations and taking in account that values reported in WW are generally lower than in DW, it becomes evident that *M. galloprovincialis* exhibits a higher accumulation capacity than *C. fluminea* and *D. rostriformis bugensis*, but lower than *D. polymorpha*. This discrepancy may be due not only to the different ionic strengths of the two environments (freshwater vs. seawater) but also to species-dependent factors. The present results are also in accordance with the study conducted by Henriques et al. (2019), in which the same mussel species showed Gd concentrations lower than 0.38 μg/g DW (below the limit of detection) after 28 days of exposure to 15 μg/L. Furthermore, at higher concentrations of 30, 60 and 120 μg/L, the species displayed concentrations of 0.44, 0.81 and 2.5 μg/g DW, respectively, confirming its bioaccumulation capacity. The present study further reflected an increased accumulation potential and BCF values at increased temperature. Similarly, the 28-day study of Mubiana and Blust (2007) with *Mytilus edulis* described a positive correlation between the accumulation of non-essential metals (cadmium and lead) and temperature. The authors attributed this trend to enhanced solubility and kinetics at higher temperatures. It was also noted that increased temperature enhanced the activity of free metal ions, which are more bioavailable. Moreover, the intensified chemical reactions and diffusion rates further facilitate uptake. In our particular case on determining the influence of temperature on Gd accumulation, it is necessary not to pinpoint a singular determining factor as bioaccumulation can also be influenced by species, tissue and experimental conditions. For instance, and unlike our observations, Figueiredo et al. (2022) recently demonstrated that Gd accumulation in the clam *Spisula solida,* following 7 days of exposure, was not influenced by water temperature. This discrepancy might stem from the different exposure durations (7 vs. 28 days). Longer exposure periods to increased temperature could induce more changes in mussels’ physiology, affecting Gd uptake rates and mechanisms for regulating its accumulation over time. Considering these divergent responses and the broader context of global warming, it is imperative to comprehensively assess organisms’ responses from the interplay between metals such as Gd and warming.

### Metabolic capacity

The difference between energy consumption and available energy reserves serves as a valuable indicator of overall organisms’ health (De Coen and Janssen 2003). Environmental modifications are known to alter organisms’ energy metabolism, reallocating energy resources toward cellular mechanisms to cope with stress rather than supporting critical functions like growth, reproduction, and development (Sokolova et al. 2012). In the current study, electron transport system (ETS) activity, considered a measure of organisms’ metabolic capacity, remained consistent across treatments despite variations in energy reserves. Specifically, Gd-exposed mussels exhibited elevated glycogen (GLY) content in comparison to the uncontaminated counterpart, irrespective of temperature. This may suggest an adaptative effort to conserve energy expenditure in the presence of Gd. A similar trend was observed in *M. galloprovincialis* exposed to Gd (15-120 μg/L), with increased GLY reserves paralleled with decreased metabolic capacity (Henriques et al. 2019; Trapasso et al. 2021). This strategy was attributed to potential physiological adjustments, including reduced filtration rate and prolonged valve closure, aimed at preventing Gd accumulation. This response was also observed in mussels exposed to the REE lanthanum (La), reinforcing the hypothesis of metabolic maintenance and/or depression under mild stress conditions (Pinto et al. 2019). Still, more research is necessary in bivalves to determine the specific mechanisms adopted to mitigate metal accumulation, such as reductions in filtration and respiration rates. The high PROT reserves exhibited in contaminated mussels at 22 °C suggests an effort to limit protein expenditure (to safeguard functional proteins' vital survival and performance). Simultaneously, an enhanced PROT synthesis may be speculated to possibly repair or replace damaged proteins. The strategy of accumulating energy reserves under stressful conditions has been previously documented in *M. galloprovincialis* at increased temperature and arsenic presence (Coppola et al. 2018), as well as in response to 17 alpha-ethinylestradiol (EE_2_) exposure coupled with elevated temperature (Lopes et al. 2022). Nevertheless, while this adaptative mechanism may compromise immediate metabolic performance, its potential long-term consequences on crucial physiological functions like reproduction and growth warrant further consideration.

### Antioxidant and biotransformation enzymes

In response to adverse environmental conditions, organisms may experience an accumulation of reactive oxygen species (ROS), prompting the activation of antioxidant defenses to mitigate ROS excess and prevent cellular damage (Regoli and Giuliani 2014). In the present study, at the control temperature, the presence of Gd increased the antioxidant capacity of mussels, suggesting that this metal stimulates the activation of this defense mechanism, possibly due to an elevation in ROS production. The enhanced antioxidant capacity was also formerly observed in *M. galloprovincialis* exposed to Gd (15-60 μg/L) (Henriques et al. 2019; Trapasso et al. 2021). In the present study, at 22 °C, Gd-exposed mussels exhibited greater antioxidant capacity compared to their non-contaminated counterpart, in line with the results observed at 17 °C between non-contaminated and Gd contaminated mussels. However, at higher temperature (22 °C), both Gd-exposed mussels and non-contaminated ones displayed reduced antioxidant capacity in comparison to those at the lowest ambient temperature (17 °C), indicating that elevated temperature alone may compromise antioxidant defenses. This trend is consistent with former findings by Andrade et al. (2022a) and Morosetti et al. (2020), who demonstrated that bivalves subjected to increased temperature or the combined effect of warming and metal contamination (e.g., La, mercury (Hg) and cerium (Ce) oxide nanoparticles) displayed diminished defense capacity.

Organisms may favor the conversion of a wide range of organic contaminants through phase I (carboxylesterases; CbEs) and/or phase II biotransformation enzymes (Glutathione *S*-transferases; GSTs), facilitating their excretion from the cells (Regoli and Giuliani 2014; Yan 2014). These enzymes, mainly involved in detoxification processes, are also susceptible to be modulated by metal contamination (Dobritzsch et al. 2020; Hauser-Davis et al. 2021). Similarly to the trend observed in antioxidant responses, mussels exposed to Gd increased their biotransformation capacity (GSTs and CbEs) in comparison to the non-contaminated counterpart. Notably, at 22 °C, both reported activities were lower than those observed at 17 °C for the non-exposed groups. Once more, Gd exposure induced the detoxification capacity in mussels (Gd-exposed vs. non-contaminated mussels), with temperature being a modulating factor (17 °C vs. 22 °C). Previous studies have already demonstrated that elevated temperature can negatively impact GSTs activity. For instance, Morosetti et al. (2020) indicated a reduction in GSTs activity in *M. galloprovincialis* mussels at increased temperature, either alone or in combination with Hg and Ce oxide nanoparticles. Similarly, Lopes et al. (2022) found decreased GSTs activity in mussels exposed to EE_2_ under increased temperature. Nevertheless, an increase of GSTs activity (which also possesses antioxidant properties) in the presence of Gd can be attributed to heightened oxidative stress derived from a rise in ROS due to metal exposure. In the same mussel species, Henriques et al. (2019) reported enhanced GSTs activity at various concentrations of Gd (ranging from 15 to 120 μg/L). The same observation was described in the digestive gland of the freshwater mussel *D. rostriformis bugensis* after 7 days of exposure to 10 μg/L of Gd (Perrat et al. 2017), further supporting the modulation of GSTs in the presence of Gd. Both studies noted that this increase in GSTs activity reached a peak and subsequently declined, potentially associated to a concentration threshold or a 28-day exposure duration, indicating a link to Gd accumulation. A plausible explanation for this phenomenon lies in the ionic radius of Gd, comparable to that of divalent Ca^2+^. Consequently, Gd may function as a calcium channel blocker (Sherry et al. 2009) as it can block Ca^2+^-dependent enzymes such as GSTs, among others (Rogosnitzky and Branch 2016). This explanation could rationalize the observed enzymatic response during co-exposure to warming and Gd. Notably, the decline in both GSTs and CbEs activities with increased temperature might compromise mussels’ defensive mechanisms, thereby amplifying their susceptibility to additional stressors.

### Oxidative damage

Lipid peroxidation (LPO) and protein carbonylation (PC) can arise when defense mechanisms are unable to effectively counteract ROS (Lushchak 2011; Pisoschi et al. 2021). In the current study, Gd was found to cause LPO, revealing that: i) at the control temperature, the efforts to prevent further Gd accumulation and the activation of antioxidant and biotransformation enzymes were insufficient; ii) at 22 °C, the ineffective defense enzymes and metabolic capacity maintenance contributed to the greatest lipid damage. Similarly, *M. galloprovincialis* exposed to concentrations ranging from 30 to 120 μg/L of Gd exhibited signs of cellular damage, regardless of the activation of several antioxidant and biotransformation enzymes (Henriques et al. 2019; Trapasso et al. 2021). In clams *C. fluminea*, LPO occurred after 7 days of exposure to 10 μg/L of Gd. The inefficiency of defense mechanisms leading to elevated LPO levels in *M. galloprovincialis* has been noted under increased temperature and various contaminants, including Hg (Coppola et al. 2017), diclofenac (Freitas et al. 2019) and rutile titanium dioxide (TiO_2_, Leite et al. 2020b). Nonetheless, neither Gd nor temperature induced protein oxidation, suggesting that lipids are more susceptible to ROS excess. While PC can result directly from amino acid side chain oxidation involving metals and hydrogen peroxide, it is also possible that PC arising indirectly from lipid-derived aldehydes is more prevalent (Grimsrud et al. 2008). Additionally, cells have mechanisms such as the induction of heat shock proteins (HSPs) in response to metal-induced and heat stress, playing a protective role in maintaining protein structure integrity (see Fabbri et al. 2008). Thus, our observation suggests that lipids are more vulnerable than proteins to Gd exposure and increased temperature. Previous studies have similarly demonstrated that *M. galloprovincialis* experienced LPO while PC levels remain stable when exposed to various contaminants such as anatase TiO_2_ (Leite et al. 2020a) and multi-walled carbon nanotubes (Andrade et al. 2019), further supporting this hypothesis.

### Neurotoxicity

The inhibition of AChE activity has been mainly used as an indicator of the toxic effects of organophosphorus and other pesticides (English and Webster 2012). However, in marine invertebrates, metals and nanoparticles can also inhibit this activity (Brown et al. 2004; De Marchi et al. 2018; Perić et al. 2017). In the present study, the presence of Gd did not result in the inhibition of AChE activity at any temperature. Also, Henriques et al. (2019) reported unchanged AChE activity under a similar Gd concentration of 15 μg/L in *M. galloprovincialis*. Yet, at higher Gd concentrations (30, 60 and 120 μg/L), AChE inhibition was observed. Nevertheless, in the present study, the rise of temperature alone led to reduced AChE activity, aligning with decreased metabolic performance (as indicated by low antioxidant and detoxifying enzymatic activities) under this condition. This observation is likely connected to the overall metabolic depression due to heat stress.

### Multivariate analysis and integrated biomarker response

The choice of a battery of biochemical parameters to evaluate the impact of chemical contaminants and other environmental stressors is highly recommended as it allows for a comprehensive and general assessment of their effects, providing a more accurate and detailed understanding of their impact on biological systems; however, the selection of multiple parameters can complicate the identification of patterns and difficult the interpretation of their relationships. Additionally, the benefits on the use of whole tissue, as opposed to specific organs, not only enables a broader spectrum of biomarkers to be evaluated and enhances cost-effectiveness, but also yields a more comprehensive and integrated understanding of the organism's overall health. To overcome drawbacks, indexes, such as IBR, aid to comprehensively assess the overall impacts of multiple stressors and simplify data interpretation. In this context, several authors have endorsed the use of the IBR model to evaluate risks in bivalves (e.g., Andrade et al. 2023; Cao et al. 2022; Chahouri et al. 2022; Khan et al. 2021). In the present study, the IBRvs2 model indicated that the rise in temperature led to depression of most biomarkers. This outcome aligns with the notion of metabolic depression of the defense enzymes analyzed, likely serving as a strategy to mitigate heat stress and prevent cellular damage. This can be further supported by the PCO analysis, which indicated stronger negative impacts on most of the biomarkers under this increased temperature. Nevertheless, organisms exposed to Gd regardless of the temperature, exhibited a lower IBRvs2 score, reflecting a contrasting trend in the deployment of defenses mechanisms. Notably, the Gd-only exposure treatment displayed a slightly higher index value, as there was an activation of the defense mechanisms due to Gd presence. In the case of organisms simultaneously exposed to Gd and increased temperature (co-exposure), the activation of defense mechanisms was countered by slight inhibition due to heat stress. In both scenarios, the IBRvs2 index was significantly affected by the elevated LPO values, particularly evident in organisms subjected to increased temperature. This observation supports the notion of Gd’s impact, especially in the context of warming. Both IBRvs2 and PCO models revealed that the defense enzymes contribution was more pronounced for Gd-exposed organisms under control conditions than in the context of increased temperature. This finding highlights how warming influences the activation of these enzymes, ultimately leading to heightened cellular damage.

## Conclusions

The consequences of Gd exposure under increased temperature on the mussel *M. galloprovincialis* were reported in here for the first time, demonstrating its toxicological effects particularly under warming conditions. Mussels exposed to Gd-alone reduced their metabolic performance possibly to prevent further Gd accumulation. However, even though certain defenses were activated, their effectiveness fell short on preventing cellular damage. Furthermore, because the defensive mechanisms were compromised at a higher temperature, the cellular damage was accentuated in Gd-exposed organisms. As a direct consequence of warming, mussels experienced metabolic depression to avoid heat stress and consequent cellular damage. This study highlights the risks that Gd can pose to coastal organisms in a global warming scenario, and consequently, the need to further investigate REEs exposures in the context of climate change-related stressors.
